# Using Image Recognition to Process Unbalanced Data in Genetic Diseases From Biobanks

**DOI:** 10.3389/fgene.2022.822117

**Published:** 2022-02-07

**Authors:** Ai-Ru Hsieh, Yi-Mei Aimee Li

**Affiliations:** Department of Statistics, Tamkang University, New Taipei, Taiwan

**Keywords:** mManhattan plot, imbalanced data, genome-wide association analyses, biobank, deep learning, image identification

## Abstract

With precision medicine as the goal, the human biobank of each country should be analyzed to determine the complete research results related to genetic diseases. In addition, with the increase in medical imaging data, automatic image processing with image recognition has been widely studied and applied in biomedicine. However, case–control data imbalance often occurs in human biobanks, which is usually solved by the statistical method SAIGE. Due to the huge amount of genetic data in human biobanks, the direct use of the SAIGE method often faces the problem of insufficient computer memory to support calculations and excessive calculation time. The other method is to use sampling to adjust the data to balance the case–control ratio, which is called Synthetic Minority Oversampling Technique (SMOTE). Our study employed the Manhattan plot and genetic disease information from the Taiwan Biobank to adjust the imbalance in the case–control ratio by SMOTE, called “TW-SMOTE.” We further used a deep learning image recognition system to identify the TW-SMOTE. We found that TW-SMOTE can achieve the same results as that of SAIGE and the UK Biobank (UKB). The processing of the technical data can be equivalent to the use of data plots with a relatively large UKB sample size and achieve the same effect as that of SAIGE in addressing data imbalance.

## 1 Introduction

As national and ethnic human databases have been established and improved in recent years, genome-wide association studies (GWAS) have become a widely used method in genetic disease research to analyze the genetics of complex diseases. In the association analysis, the Manhattan plot is a visual representation of the *p*-value position of a single nucleotide polymorphism (SNP) association (Jain et al., 1999).

As the technology of precision medicine continues to evolve, more and more researchers are using human biobanks, but researchers usually look for large and easily accessible human biobanks for their research. However, depending on the ethnicity or even country, genetic diseases may be attributed to different genetic and environmental factors. Therefore, with the goal of precision medicine, the human biobanks in each country should be analyzed to determine complete genetic disease–related research results.

Case–control data imbalance often occurs in human biobanks, which is usually addressed by the statistical method SAIGE (Zhou et al., 2018), as it uses generalized mixed model association testing to correct data imbalance in association analysis. Due to the huge amount of genetic data in the human biobanks, the direct use of the SAIGE method often faces the problem of insufficient computer memory to support the computation, and the computation time is too long. In the case of extreme case–control imbalance in the database, another common method is to use sampling to adjust the data to balance the case–control ratio, which is called the Synthetic Minority Oversampling Technique (SMOTE). It has been shown that the SMOTE method can improve the classification accuracy of a few categories (Chawla et al., 2002).

In addition, with the increase of medical imaging data, automatic image processing with image recognition has been widely studied and applied in biomedicine (Smistad et al., 2015). Preprocessing of medical images includes histogram equalization, smoothing, erosion, and dilation. These techniques have been combined to develop a medical image processing library, which is widely used to identify diseases and determine whether or not the organs are normal (Widodo et al., 2020). The results of the GWAS analysis are presented through a Manhattan plot, where the SNP-associated *p*-value was used as an image feature to match the similarity with other Manhattan images using a deep learning training model. This analysis method improves the performance and speed of computing when matching a large database.

This study employed the Manhattan plot and genetic disease information from the Taiwan Biobank (TWB) to correct the information imbalance after the same information was treated by SMOTE, and this statistical analysis result was the same as that of SAIGE. Furthermore, we used a deep learning image recognition system to identify the TWB with relatively few subjects, in order to generate data with SMOTE, which can achieve the same results as the UK Biobank (UKB), which has more subjects, to explain genetic diseases.

## 2 Materials and Methods

### 2.1 Study Population

The participants and their data were obtained exclusively from the TWB (https://www.twbiobank.org.tw/test_en) (Wei et al., 2021). Up to April 15th 2021, more than 144,000 participants had been recruited. The demographic and health-related survey data for the 105,388 study subjects were released in December 2019.

### 2.2 Gentyping, Quality Control, and GWAS

Detailed genotyping and imputation procedures have been described by Wei et al. (2021). The 27,604 subjects and 632,172 SNPs were genotyped with the customized TWB1 array in this study. We first homogenized the controls by removing comorbid individuals from the control group of each trait. Comorbid diseases are defined by a data-driven method using the partitioning around medoids (PAM) (Van der Laan and Pollard, 2003; Zhang and Couloigner, 2005; Schubert and Rousseeuw, 2019) algorithm in the cluster package of R (version 3.6) and φ-correlation as our distance matrices. The best-fit group numbers were selected by maximizing the silhouette score (Rousseeuw, 1987).

Subjects and SNPs were extracted by the following criteria: 1) call rate > 0.95; 2) MAF > 0.01; and 3) deviation from Hardy–Weinberg equilibrium, *p* > 0.001. The QC and GWAS analyses were performed using PLINK2 (https://www.cog-genomics.org/plink/2.0).

### 2.3 Data Imbalance Processing, SMOTE

The basic principle of SMOTE is to select a sample from a small number of samples as the basis for generating a new sample, and then, randomly select a sample as its auxiliary sample from the k neighboring samples of the same category according to sample multiplicity n, and repeat the above n times. Then, n final samples are generated for the samples and the auxiliary samples.

$$x_{new,attr}=x_{i,attr}+\left(x_{ij,attr}-x_{i,attr}\right)\times \gamma$$

wherein $X_{i}\in R^{d},\;x_{i,attr}$ represents the attr-th attribute of the *i*-th sample of the minority class, attr = 1, 2, 3, d; γ denotes a random variable between [0,1] and the *j*-th neighboring sample of the $x_{ij}$ sample $x_{i}$ , j = 1, 2, 3, …, k; $x_{new}$ is the final new sample generated by the difference between $x_{ij}\;\text{and}\;x_{i}$ (Chawla et al., 2002).

In order to determine which parameter value would make the TW-SMOTE–adjusted Manhattan plot the most compatible with the TW-SAIGE–adjusted Manhattan plot in Taiwan, this study compared the most appropriate ratio of the generated data for disease according to the scales of 0.1, 0.03, 0.005, and 0.001.

### 2.4 Image Recognition

This study divided image recognition into three items: 1) TW-SMOTE: TWB used SMOTE to deal with data imbalance; 2) TW-SAIGE: TWB used SAIGE to deal with data imbalance; and 3) UK-SAIGE: UKB used SAIGE to deal with data imbalance.

### 2.4.1 Image-Based Smoothing and Morphological Manipulation

This study used the open source Computer Vision library (OpenCV) in Python to extract features by removing outliers and noise (Bradski and Kaehler, 2000) while preserving the Manhattan graph information. OpenCV uses the morphological operations of Dilation and Erosion to identify the very large and very small areas in an image. Dilation is similar to “field expansion,” which expands the highlighted areas or white parts of an image, and the resulting image is larger than the highlighted areas of the original image; Erosion is similar to “field erosion,” which shrinks the highlighted areas or white parts of the image, meaning the highlighted or white part of the image is reduced and refined, and the resulting image is smaller than the highlighted area of the original image. Finally, this study used the gradient operation, where the gradient operation is equal to dilation–erosion (Mordvintsev and Abid, 2014).

#### 2.4.2 Building an Image Classification Model

This study used a Convolutional Neural Network (CNN) to construct a deep learning model using TensorFlow and Keras (Albawi et al., 2017). The CNN in this study has three convolutional layers (including ReLu and Max Pooling2D), where each convolutional layer is convolved with 3 × 3 filters, and the three layers extract 32, 32, and 64 filters, respectively. The final output is obtained by adding the softmax function to the last node, and the value ranges from 0.0 to 1.0 (O'Shea and Nash, 2015).

**FIGURE 1:**
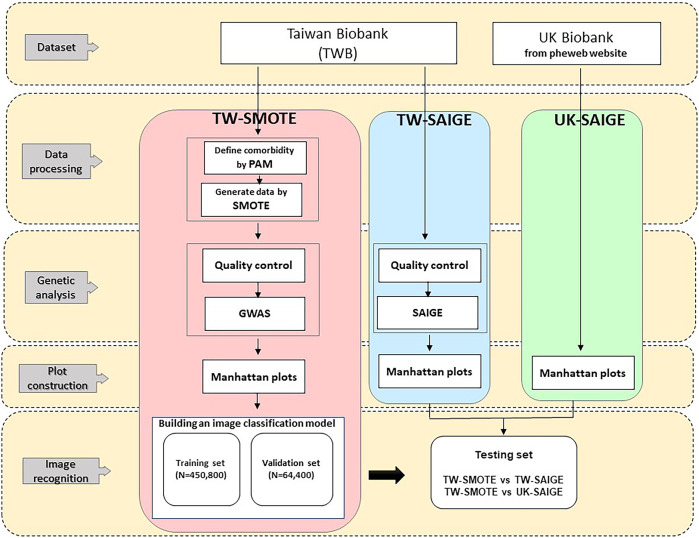
Analysis flow of this study.

In order to limit the training set, this study boosted the data by a series of transformations, meaning the model would not see two identical pictures, which helped to suppress model overfitting and enhanced the model's predictive capacity. This study implemented Keras, which uses keras.preprocessing.image.ImageDataGenerator, epochs = 20, and batch_size = 32. The analysis flow of this study is shown in Figure 1. The models were trained according to the above parameters, and model prediction was performed on the testing set (Chollet, 2016). For each trait, there were 515,200 Manhattan plots for building an image classification model in our study. Among them, we used 85% of the Manhattan plots for training and 15% for validation. As three disease categories [hypertension (HPT), asthma (AST), irritable bowel syndrome (IBS)] were used in this study, three similarity values were assigned to one Manhattan plot for each prediction, which were summed to 1. The similarities between the Manhattan plot and the corresponding three diseases are represented by the model classification, where the highest of the three similarity values was used as the basis for model classification. In this study, the loss which is equal to the distance between the real and predicted, and accuracy which is equal to the number of correct classifications/the total number of classifications were used as the indicators of model performance in the training set, and similarity was used as the indicator of model performance evaluation in the testing set (Lee and Song, 2019).

## 3 Results

This study divided the data analysis into four parts. The first part conducted data cleaning before data analysis, which included disease clustering to remove comorbidities and data quality control using PLINK2. The second part performed TW-SAIGE and TW-SMOTE imbalance data processing. The third part built a deep learning image recognition model using the TW-SMOTE and TW-SAIGE Manhattan data sets and adjusted the TW-SMOTE–related parameters according to their image recognition model training results in order to fulfill the objectives of this study. The fourth part performed sequential image recognition on UK-SAIGE.

### 3.1 Partitioning Around Medoid

Before drawing a Manhattan plot and performing image recognition, the data must be preprocessed to complete data cleaning and correction in several steps according to the nature of the data. As one of the steps is to remove the comorbidities of the target diseases to avoid bias in analysis, this study clustered diseases to identify the possible comorbidities. Before using the PAM, only 23 diseases with high prevalence in TWB were included in the clustering. The PAM was set to k = 8; the comorbidities were divided into eight groups, and the cosine similarity distance was used to calculate the clustering. The results are shown in Supplementary Figure S1.

### 3.2 Data Imbalance Processing

Among the 23 diseases, three were selected from the different subgroups of the PAM: AST, HPT, and IBS. Based on the above PAM subgroups (Supplementary Figure S1), we removed subjects with the same subgroup of diseases for each of the three diseases, and 2,542 subjects with AST, 3,252 subjects with HPT, and 7,058 subjects with IBS were excluded.

**FIGURE 2:**
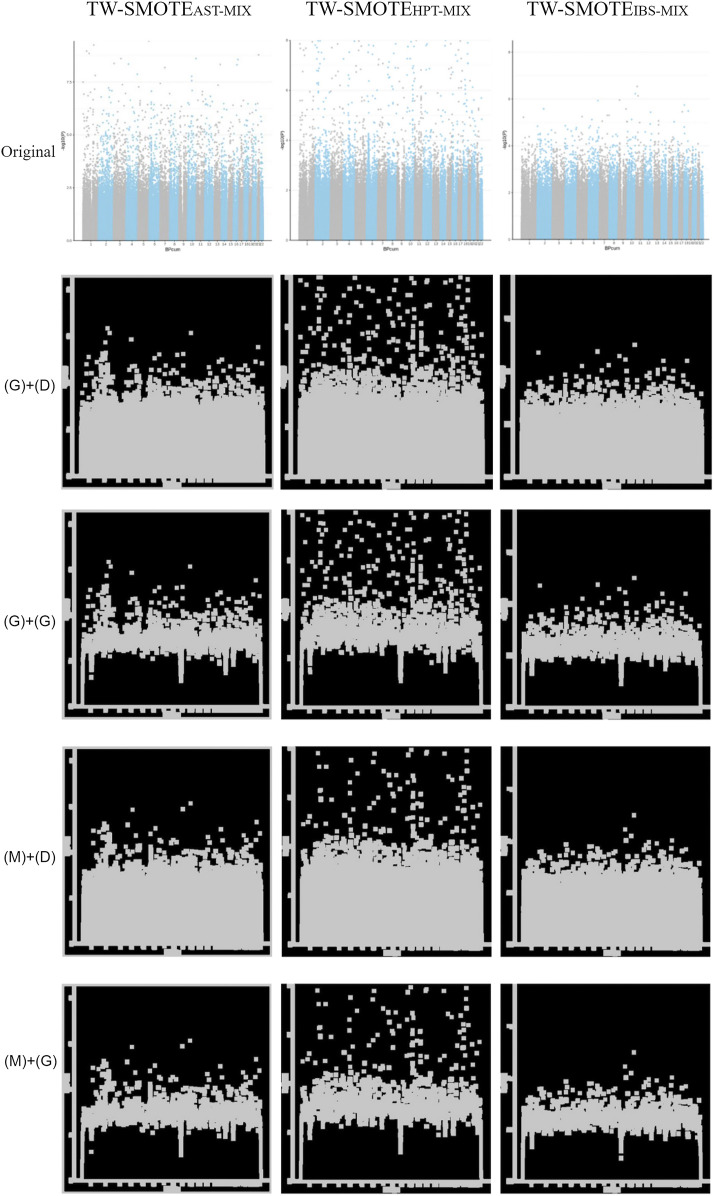
Parameters for modifying the SMOTE adjustment ratios were determined using a mixture setting with TW-SMOTE_AST-MIX_, TW-SMOTE_HPT-MIX_, and TW-SMOTE_IBS-MIX_ (AST = 0.001, HPT = 0.005, and IBS = 0.03) in “original” labeled, Gaussian filter (G) + dilation (D), Gaussian filter (G) + morphological gradient operation (G), median filter (M) + dilation (D), and median filter (M) + morphological gradient operation (G).

**FIGURE 3:**
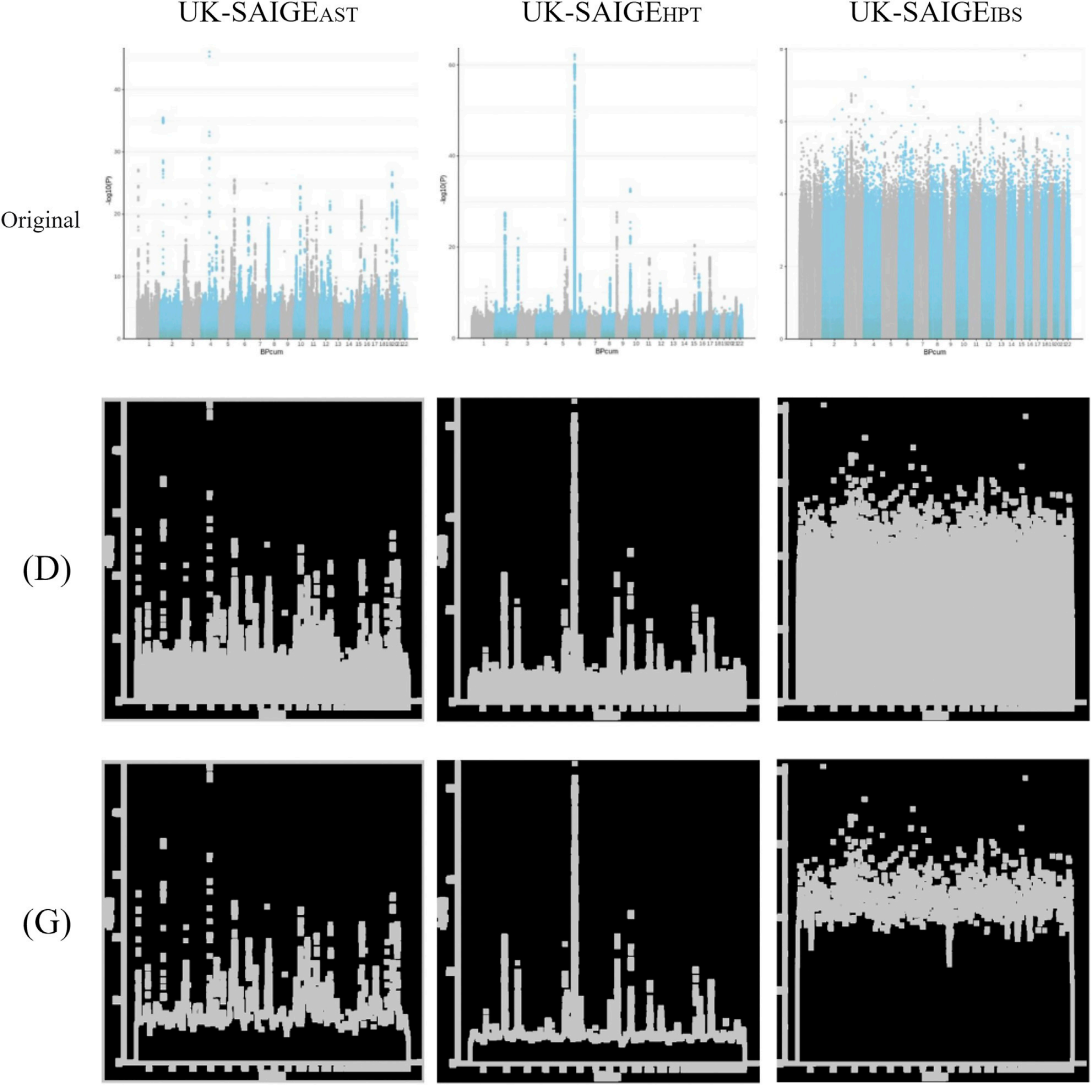
Unprocessed Manhattan plots of UK-SAIGE_AST_, UK-SAIGE_HPT_, and UK-SAIGE_IBS_ (“original” labeled) were adjusted by image processing for dilation [(D) labeled] and morphological gradient calculation [(G) labeled].

**FIGURE 4:**
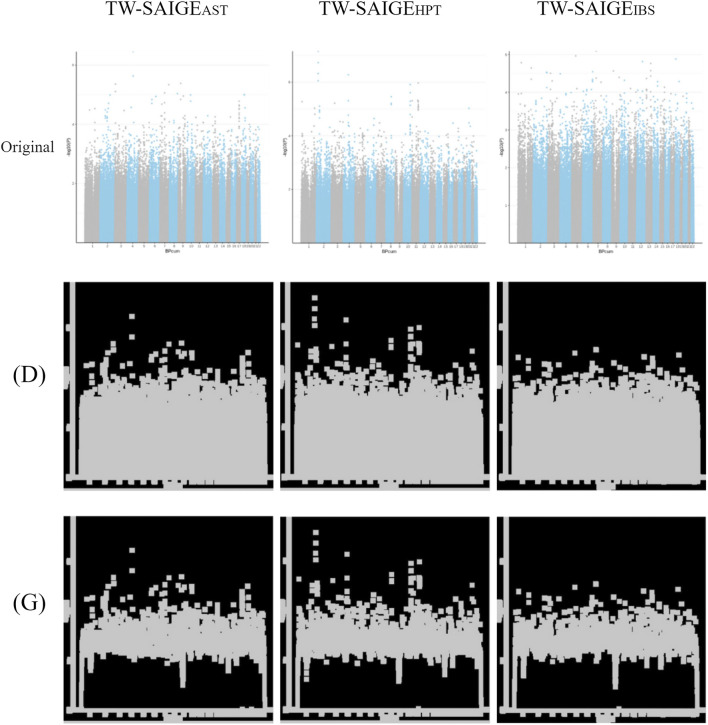
Unprocessed Manhattan plots of TW-SAIGE_AST_, TW-SAIGE_HPT_, and TW-SAIGE_IBS_ (“original” labeled) were adjusted by image processing for dilation [(D) labeled] and morphological gradient calculation [(G) labeled].

Since age and gender are common variables affecting disease occurrence, stratification by age and gender can improve the appropriateness of data generation. This study divided the data into eight groups according to gender and age percentile (25%, 47 years; 50%, 55 years; and 75%, 60 years). After QC, the Manhattan plot results of the association analysis are presented in Figure 2, Figure 3, and Figure 4 in “original” labeled.

### 3.3 Image Recognition

#### 3.3.1 UK-SAIGE

The unprocessed Manhattan plot of UK-SAIGE (Figure 3 in “original” labeled) was adjusted by image processing for dilation [Figure 3 in “(D)” labeled] and morphological gradient calculation [Figure 3 in “(G)” labeled].

#### 3.3.2 TW-SAIGE

After QC by the PAM, excluding comorbidities and SNP, the Manhattan plot results of SAIGE are presented in Figure 4 in “original” labeled. After image processing, the Manhattan plot was adjusted by dilation [Figure 4 in “(D)” labeled] and morphological gradient calculation [Figure 4 in “(G)” labeled].

#### 3.3.3 TW-SMOTE

There are four combinations of image processing according to the filters (Gaussian filter, median filter) and morphology (dilation, morphological gradient operation): Gaussian filter (G) + dilation (D), Gaussian filter (G) + morphological gradient operation (G), median filter (M) + dilation (D), and median filter (M) + morphological gradient operation (G).

##### 3.3.3.1 Adjusting the Ratio to 0.005

In the Gaussian filter (G) + dilation (D), the Gaussian filter could partially remove the outliers and noise, and it was found that the Manhattan plot of TW-SMOTE_HPT-GD-0.005_ [Supplementary Figure S2 in “(G)+(D)” labeled] was already similar to that of TW-SAIGE_HPT-D_ [Figure 4 in “(D)” labeled]. However, the TW-SMOTE_IBS-GD-0.005_ [Supplementary Figure S2 in “(G)+(D)” labeled] and TW-SAIGE_IBS-D_ [Figure 4 in “(D)” labeled] signals (Manhattan Y-value) were low, while the TW-SMOTE_AST-GD-0.005_ [Supplementary Figure S2 in “(G)+(D)” labeled] and TW-SAIGE_AST-D_ [Figure 4 in “(D)” labeled] signals (Manhattan y-axis) were high. In the training set, the best performance was obtained with validation_steps = 800 and steps_per_epoch = 3 (loss = 0.0061, accuracy = 1, Supplementary Table S1). In the testing set, when validation_steps = 400 and 800, the best prediction result was obtained (4/15 correct predictions). Among them, AST performed the best at validation_steps = 400, and TW-SMOTE_AST-GD-0.005_ was correctly predicted three out of five times [similarity = 0.3406 (steps_per_epoch = 5) to 0.7259 (steps_per_epoch = 2)]. HPT performed the best at validation_steps = 800, and TW-SMOTE_HPT-GD-0.005_ was correctly predicted four out of five times [similarity = 0.3388 (steps_per_epoch = 4) to 0.8618 (steps_per_epoch = 3)].

In the Gaussian filter (G) + morphological gradient operation (G), the Gaussian filter could remove the outliers and noise part [Supplementary Figure S2 in “(G)+(G)” labeled]. In the training set, when validation_steps = 400 and steps_per_epoch = 3, the model showed the best performance (loss = 0.0036, accuracy = 1, Supplementary Table S1). In the testing set, the best prediction result was obtained at validation_steps = 4, 400, and 800 (5/15 correct predictions). Among them, AST performed best at validation_steps = 4, 400, and 800, and TW-SMOTE_AST-GG-0.005_ was correctly predicted once out of the five times [similarity = 0.3478 (validation_steps = 400, steps_per_epoch = 4) to 0.4679 (validation_steps = 4, steps_per_epoch = 4)]. While HPT performed well in all settings of validation_steps, TW-SMOTE_HPT-GG-0.005_ was correctly predicted four out of five times [similarity = 0.5981 (validation_steps = 800, steps_per_epoch = 1) to 0.9999 (validation_steps = 400, steps_per_epoch = 3)].

In the median filter (M) + dilation (D), after filtering out the noise floating above the Manhattan plot, TW-SMOTE_IBS-MD-0.005_ and TW-SMOTE_AST-MD-0.005_ [Supplementary Figure S2 in “(M)+(D)” labeled] were slightly different to those of TW-SAIGE_IBS-D_ and TW- SAIGE_AST-D_ [Figure 4 in “(D)” labeled]. However, TW-SMOTE_HPT-MD-0.005_ was still unable to solve the problem of too much noise using median filtering. In the training set, the best performance was obtained at validation_steps = 40 and steps_per_epoch = 3 (loss = 0.0055, accuracy = 1, Supplementary Table S1). In the testing set, the best prediction result was obtained at validation_steps = 40 (5/15 correct predictions). AST performed the best with validation_steps = 4 and 40, and TW-SMOTE_AST-MD-0.005_ was correctly predicted twice out of the five times [similarity = 0.5552 (validation_steps = 4, steps_per_epoch = 3) to 0.9319 (validation_steps = 4, steps_per_epoch = 2)]. HPT performed the best at validation_steps = 40, and TW-SMOTE_HPT-MD-0.005_ was correctly predicted in three out of five predictions [similarity = 0.3533 (steps_per_epoch = 4) to 0.63334 (steps_per_epoch = 1)]. For IBS with validation_steps = 800, TW-SMOTE_IBS-MD-0.005_ had successful recognition similar to TW-SAIGE_IBS_ [similarity = 0.3544 (validation_steps = 800, steps_per_epoch = 5)].

In the median filter (M) + morphological gradient operation (G), the image features were divided into the upper and lower sawtooth patterns [Supplementary Figure S2 in “(M)+(G)” labeled]. In the training set, the best performance was obtained with validation_steps = 400 and steps_per_epoch = 3 (loss = 0.0011, accuracy = 1, Supplementary Table S1). In the testing set, the best prediction result was obtained at validation_steps = 40 (5/15 correct predictions). AST performed the best at validation_steps = 40, and TW-SMOTE_AST-MG-0.005_ was correctly predicted once out of the five times [similarity = 0.3476 (steps_per_epoch = 4)]. HPT performed the best at validation_steps = 4, 40, and 400, and TW-SMOTE_HPT-MG-0.005_ was correctly predicted four out of five times [similarity = 0.3564 (validation_steps = 400, steps_per_epoch = 5) to 0.9999 (validation_steps = 4, steps_per_epoch = 3)]. When validation_steps = 800, TW-SMOTE_IBS-MG-0.005_ had a successful recognition similar to TW-SAIGE_IBS-G_ [similarity = 0.4319 (validation_steps = 800, steps_per_epoch = 4), Supplementary Figure S2 in “(M)+(G)” labeled, Figure 4 in “(G)” labeled].

The difference between the Manhattan plots of TW-SMOTE_IBS_ and TW-SMOTE_AST_ was large when the SMOTE-generated data was adjusted to 0.005. However, the TW-SMOTE_IBS_ (Supplementary Figure S2) and TW-SAIGE_IBS_ (Figure 4) signals (Manhattan y-axis) were low, while the TW-SMOTE_AST_ (Supplementary Figure S2) and TW-SAIGE_AST_ (Figure 4) signals (Manhattan y-axis) were high.

##### 3.3.3.2 Adjusting the Ratio to 0.03

In the Gaussian filter (G) + dilation (D), there was still a lot of noise above the Manhattan plot [Supplementary Figure S3 in “(G)+(D)” labeled]. In the training set, when validation_steps = 400 and steps_per_epoch = 3, the model showed the best performance (loss = 0.1906, accuracy = 1, Supplementary Table S2). In the testing set, the best prediction result was obtained at validation_steps = 800 (6/15 correct predictions). Among them, AST performed the best at validation_steps = 40 and 800, and TW-SMOTE_AST-GD-0.03_ was correctly predicted twice out of the five times [similarity = 0.4170 (validation_steps = 800, steps_per_epoch = 1) to 0.7340 (validation_steps = 40, steps_per_epoch = 2)]. HPT performed the best at validation_steps = 4 and 400 and was correctly predicted three out of five times for TW-SMOTE_HPT-GD-0.03_ [similarity = 0.3822 (validation_steps = 4, steps_per_epoch = 4) to 0.8125 (validation_steps = 400, steps_per_epoch = 3)]. IBS performed the best at validation_steps = 800, and TW-SMOTE_IBS-GD-0.03_ was correctly predicted twice out of the five times [similarity = 0.3509 (steps_per_epoch = 5) to 0.4404 (steps_per_epoch = 4)].

In the Gaussian filter (G) + morphological gradient operation (G), the Gaussian filter still retained more noise [Supplementary Figure S3 in “(G)+(G)” labeled]. In the training set, at validation_steps = 4, steps_per_epoch = 2, the model showed the best performance (loss = 0.1403, accuracy = 0.9666, Supplementary Table S2). In the testing set, the best prediction results were obtained at validation_steps = 4, 400, and 800 (5/15 correct predictions). Among them, AST performed the best at validation_steps = 40 and 400, TW-SMOTE_AST-GG-0.03_ four out of five predictions were correct [similarity = 0.3503 (validation_steps = 400, steps_per_epoch = 4) to 0.6586 (validation_steps = 400, steps_per_epoch = 3)]. HPT performed the best at validation_steps = 4 and 800 and was correctly predicted twice by TW-SMOTE_HPT-GG-0.03_ out of the five predictions [similarity = 0.3462 (validation_steps = 4, steps_per_epoch = 4) to 0.8761 (validation_steps = 4, steps_per_epoch = 3)]. IBS performed the best with validation_steps = 4, and TW-SMOTE_IBS-GG-0.03_ was predicted correctly once out of the five times (similarity = 0.3450, steps_per_epoch = 5).

In the median filter (M) + dilation (D), it was found that the noise floating above the Manhattan plot could be filtered out [Supplementary Figure S3 in “(M)+(D)” labeled]. In the training set, with validation_steps = 400 and steps_per_epoch = 3, the model showed the best performance (loss = 0.0214, accuracy = 1, Supplementary Table S2). In the testing set, validation_steps performed fairly well in each setting (all 5/15 correct predictions). Among them, AST performed the best at validation_steps = 40, 400, and 800, and TW-SMOTE_AST-MD-0.03_ was correctly predicted three out of five times [similarity = 0.3524 (validation_steps = 400, steps_per_epoch = 4) to 0.7946 (validation_steps = 400, steps_per_epoch = 3)]. HPT performed the best at validation_steps = 4, 40, and 400 and was correctly predicted twice by TW-SMOTE_HPT-MD-0.03_ out of the five predictions [similarity = 0.3782 (validation_steps = 400, steps_per_epoch = 1) to 0.9502 (validation_steps = 400, steps_per_epoch = 1)]. IBS performed the best with validation_steps = 4 and 800, and TW-SMOTE_IBS-MD-0.03_ was predicted correctly once out of the five predictions [similarity = 0.3413 (validation_steps = 4, steps_per_epoch = 5) to 0.3528 (validation_steps = 800, steps_per_epoch = 4)].

In the median filter (M) + morphological gradient operation (G), the image features were divided into upper and lower sawtooth patterns [Supplementary Figure S3 in “(M)+(G)” labeled], and its performance was not as good as that of the dilation operation image processing. In the training set, the best performance was obtained at validation_steps = 40 and steps_per_epoch = 3 (loss = 0.1821, accuracy = 0.9622, Supplementary Table S2). In the testing set, the best prediction result was obtained at validation_steps = 800 (6/15 correct predictions). Among them, AST performed the best at validation_steps = 4 and 400, and TW-SMOTE_AST-MG-0.03_ was correctly predicted twice out of the five times [similarity = 0.3406 (validation_steps = 400, steps_per_epoch = 5) to 0.6919 (validation_steps = 400, steps_per_epoch = 3)]. HPT performed the best at validation_steps = 800, and TW-SMOTE_HPT-MG-0.03_ was predicted correctly in each of the five predictions [similarity = 0.3847 (steps_per_epoch = 4) to 0.8618 (steps_per_epoch = 2)]. IBS performed the best at validation_steps = 40, and TW-SMOTE_IBS-MG-0.03_ was predicted correctly once out of the five predictions (similarity = 0.3508, steps_per_epoch = 5).

TW-SMOTE_IBS_ and TW-SMOTE_AST_ were already similar to the Manhattan plot of TW-SAIGE when the data was scaled to 0.03. After image processing, we can see that the median filtered image had less signal than the Gaussian filtered image; however, with the TW-SMOTE_HPT_ generated data scaled to 0.03, the message point (y-axis of the Manhattan plot) was much lower. However, the similarity between the Manhattan plots of TW-SMOTE_HPT_ and TW-SAIGE_HPT_ was still a bit different. In addition, as the median filter (M) retained fewer features than the Gaussian filter (G), it generated a worse classification effect than other image processing combinations.

##### 3.3.3.3 Adjusting the Ratio to 0.1

In the Gaussian filter (G) + dilation (D), as the eigenstyles of AST and HPT were both high, we may only judge them according to the jagged height above the Manhattan plot [Supplementary Figure S4 in “(G)+(D)” labeled]. In the training set, the model performed best when validation_steps = 4 and steps_per_epoch = 3 (loss = 0.3079, accuracy = 0.8591, Supplementary Table S3). In the testing set, the best prediction result was obtained at validation_steps = 4 and 800 (5/15 correct predictions). Among them, AST performed the best at validation_steps = 40 and 400, and TW-SMOTE_AST-GD-0.1_ was correctly predicted three out of five times [similarity = 0.3448 (validation_steps = 400, steps_per_epoch = 4) to 0.7300 (validation_steps = 40, steps_per_epoch = 3)]. HPT performed the best at validation_steps = 4 and was correctly predicted three out of five times for TW-SMOTE_HPT-GD-0.1_ [similarity = 0.3583 (steps_per_epoch = 5) to 0.7300 (steps_per_epoch = 3)]. IBS performed the best at validation_steps = 800, and TW-SMOTE_IBS-GD-0.1_ was correctly predicted twice out of the five times [similarity = 0.3372 (steps_per_epoch = 5) to 0.3573 (steps_per_epoch = 4)].

In the Gaussian filter (G) + morphological gradient operation (G), the features were divided into upper and lower sawtooth patterns [Supplementary Figure S4 in “(G)+(G)” labeled]. The model performed the best in the training set with validation_steps = 800 and steps_per_epoch = 3 (loss = 0.0521, accuracy = 1, Supplementary Table S3). In the testing set, the best prediction result was obtained at validation_steps = 400 (6/15 correct predictions). Among them, AST performed the best at validation_steps = 4 and 40, and TW-SMOTE_AST-GG-0.1_ was correctly predicted three out of five times [similarity = 0.3442 (validation_steps = 4, steps_per_epoch = 4)] to 0.8502 (validation_steps = 40, steps_per_epoch = 2)]. HPT performed the best at validation_steps = 400 and was correctly predicted three out of five times by TW-SMOTE_HPT-GG-0.1_ [similarity = 0.3618 (steps_per_epoch = 1) to 0.8850 (steps_per_epoch = 3)]. IBS performed the best at validation_steps = 40, 400, and 800, with TW-SMOTE_IBS-GG-0.1_ being correct in one out of the five predictions [similarity = 0.3474 (validation_steps = 800, steps_per_epoch = 4) to 0.3626 (validation_steps = 400, steps_per_epoch = 5)].

In the median filter (M) + dilation (D), as the eigenstyles were all high, they could only be judged according to the jagged height above the Manhattan plot [Supplementary Figure S4 in “(M)+(D)” labeled]. In the training set, the best performance was obtained when validation_steps = 40 and steps_per_epoch = 3 (loss = 0.0516, accuracy = 0.9677, Supplementary Table S3). In the testing set, the best performance was obtained at validation_steps = 400 (6/15 correct predictions). Among them, AST performed the best at validation_steps = 400, and TW-SMOTE_AST-MD-0.1_ was correctly predicted four out of five times [similarity = 0.3786 (steps_per_epoch = 5) to 0.6191 (steps_per_epoch = 3)]. HPT performed the best at validation_steps = 40 and 800 and was correctly predicted twice by TW-SMOTE_HPT-MD-0.1_ in five predictions [similarity = 0.3753 (validation_steps = 40, steps_per_epoch = 4) to 0.7045 (validation_steps = 800, steps_per_epoch = 2)]. IBS performed the best at validation_steps = 4, and TW-SMOTE_IBS-MD-0.1_ was predicted correctly one out of five times [similarity = 0.3464 (steps_per_epoch = 4) to 0.3469 (steps_per_epoch = 5)].

In the median filter (M) + morphological gradient operation (G), the features were divided into upper and lower sawtooth patterns, but fewer features were retained, as compared to other graphical processing methods [Supplementary Figure S4 in “(M)+(G)” labeled]. The model performed the best in the training set with validation_steps = 40 and steps_per_epoch = 2 (loss = 0.2135, accuracy = 0.9375, Supplementary Table S3). In the testing set, validation_steps = 40 showed the best prediction result (6/15 correct prediction). Among them, AST performed the best at validation_steps = 40, and TW-SMOTE_AST-MG-0.1_ was correctly predicted twice out of the five times [similarity = 0.3620 (steps_per_epoch = 5) to 0.4064 (steps_per_epoch = 1)]. HPT performed the best at validation_steps = 40, and TW-SMOTE_HPT-MG-0.1_ was correctly predicted twice out of the five times [similarity = 0.3459 (steps_per_epoch = 4) to 0.7886 (steps_per_epoch = 3)]. IBS performed the best at validation_steps = 4 and 400, and TW-SMOTE_IBS-MG-0.1_ was correctly predicted in one out of five predictions [similarity = 0.3536 (validation_steps = 4, steps_per_epoch = 4) to 0.3617 (validation_steps = 400, steps_per_epoch = 5)].

When the HPT ratio was adjusted to 0.1, the TW-SMOTE_HPT_ statistically significant signal was spread over almost the entire Manhattan plot, especially the Gaussian filter (G) + dilation (D) [Supplementary Figure S4 in “(G)+(D)” labeled], as Gaussian filtering preserves relatively more image features. However, the drawback is that dilation (D) increases the signal of these noise points (outliers) (Supplementary Figure S4). As the difference between Manhattan plot characteristics of TW-SMOTE_HPT_ and the TW-SAIGE_HPT_ increased, it increased the error rate of image recognition.

### 3.3.3.4 Mixing Ratio

According to the findings in Sections 3.4.3.1 to 3.4.3.3, the parameters for modifying the SMOTE adjustment ratios were determined using a mixture setting with AST = 0.001, HPT = 0.005, and IBS = 0.03. As can be seen in Figure 2, TW-SMOTE_AST-MIX_, TW-SMOTE_HPT-MIX_, and TW-SMOTE_IBS-MIX_ were found to have similar characteristics, as compared to the TW-SAIGE Manhattan plot.

The model had the best performance in the training set of Gaussian filter (G) + dilation (D) with validation_steps = 4 and steps_per_epoch = 3 [loss = 0.0046, accuracy = 1, Supplementary Table S4, Figure 2 in “(G)+(D)” labeled]. In the testing set, validation_steps = 800 had the best prediction result (12/15 correct predictions). Among the three diseases, TW-SMOTE_HPT-GD-MIX_ showed the best performance (17/20 correct predictions) with similarity = 0.3560 (validation_steps = 800, steps_per_epoch = 4) to 0.9997 (validation_steps = 800, steps_per_epoch = 3). TW-SMOTE_IBS-GD-MIX_ performed second best (15/20 correct predictions), with similarity = 0.3440 (validation_steps = 4, steps_per_epoch = 4) to 0.9999 (validation_steps = 40, steps_per_epoch = 3 and 4). TW-SMOTE_AST-GD-MIX_ performed poorly (11/20 correct predictions), similarity = 0.3812 (validation_steps = 400, steps_per_epoch = 5) to 0.9988 (validation_steps = 400, steps_per_epoch = 3).

The model had the best performance in the training set of Gaussian filter (G) + morphological gradient operation (G) with validation_steps = 800 and steps_per_epoch = 3 [loss = 0.0066, accuracy = 1, Supplementary Table S4, Figure 2 in “(G)+(G)” labeled]. In the testing set, validation_steps = 400 had the best prediction (12/15 correct predictions). Among the three diseases, TW-SMOTE_HPT-GG-MIX_ had the best performance (17/20 correct predictions) with similarity = 0.3614 (validation_steps = 400, steps_per_epoch = 4) to 0.9999 (validation_steps = 800, steps_per_epoch = 3). TW-SMOTE_IBS-GG-MIX_ was the second (14/20 correct predictions), similarity = 0.3031 (validation_steps = 800, steps_per_epoch = 4) to 0.9991 (validation_steps = 800, steps_per_epoch = 3). TW-SMOTE_AST-GG-MIX_ performed the worst (10/20 correct predictions), similarity = 0.3522 (validation_steps = 4, steps_per_epoch = 4) to 0.9469 (validation_steps = 40, steps_per_epoch = 3).

The model had the best performance in the training set of median filter (M) + dilation (D) with validation_steps = 400 and steps_per_epoch = 3 [loss = 0.0016, accuracy = 1, Supplementary Table S4, Figure 2 in “(M)+(D)” labeled]. In the testing set, validation_steps = 4 and 400 showed the best prediction results (10/15 correct predictions). Among the three diseases, TW-SMOTE_HPT-MD-MIX_ and TW-SMOTE_IBS-MD-MIX_ had the best performances (14/20 correct predictions). TW-SMOTE_AST-MD-MIX_ performed the worst (10/20 correct predictions), with similarity = 0.3670 (validation_steps = 40, steps_per_epoch = 5) to 0.9871 (validation_steps = 4, steps_per_epoch = 2).

The model had the best performance in the training set of median filter (M) + morphological gradient operation (G) with validation_steps = 40 and steps_per_epoch = 3 [loss = 0.0131, accuracy = 0.9944, Supplementary Table S4, Figure 2 in “(M)+(G)” labeled]. In the testing set, validation_steps = 400 had the best prediction result (12/15 correct predictions). The performance of TW-SMOTE_HPT-MG-MIX_, TW-SMOTE_IBS-MG-MIX_, and TW-SMOTE_AST-MG-MIX_ were comparable (both 13/20 correct predictions). TW-SMOTE_HPT-MG-MIX_ performed the best at validation_steps = 800, and each of the five predictions were predicted correctly [similarity = 0.3746 (steps_per_epoch = 4) to 0.9999 (steps_per_epoch = 3)]. TW-SMOTE_IBS-MG-MIX_ also performed the best at validation_steps = 800, with four out of five predictions being correct [similarity = 0.3151 (steps_per_epoch = 4) to 0.9999 (steps_per_epoch = 3)]. TW-SMOTE_AST-MG-MIX_ also performed the best at validation_steps = 4, with four out of five predictions being correct [similarity = 0.3474 (steps_per_epoch = 4) to 0.9906 (steps_per_epoch = 3)].

Table S4 and Figure 2 show that median filtering can help remove noise and preserve the features of the Manhattan plot in most cases. In addition, if the Manhattan features are enhanced through the dilation operation, in most cases, the classification basis of the recognition system of the training model would not be confused. In addition, the training model generally performed the best (the lowest loss and highest accuracy) when the parameter steps_per_epoch = 2 or 3 was set. The training model generally performed better when validation_steps = 4 (the lowest loss and highest accuracy), which can reduce the computing time of the training model and generate good image recognition results.

### 3.4 UK-SAIGE Image Recognition

This study adopted the trained image recognition model combination: TW-SMOTE with mixed ratio of median filter (M) + dilation (D) [Figure 2 in “(M)+(D)” labeled], the model parameters of steps_per_epoch = 2 and validation_steps = 4 to train the model, and UK-SAIGE Manhattan plot + dilation (D) [Figure 3 in “(D)” labeled] to predict the model. According to the graph of correctness and the loss rate of the model, the correctness rate increased and the error rate decreased as the number of training iterations was increased. After 20 training iterations, the model reached a correct rate of more than 90%. TW-SMOTE_MD-MIX_ was compared with UK-SAIGE Manhattan plot + morphological gradient operation (G) [(Figure 3 in “(G)” labeled] for image recognition, with TW-SMOTE_HPT-MD-MIX_ (similarity = 0.9719, Supplementary Table S5) and TW-SMOTE_IBS-MD-MIX_ (similarity = 0.6197, Supplementary Table S5).

## 4 Discussion and Conclusion

The GWAS results of TWB are illustrated via a Manhattan plot, where the *p*-value of the SNP loci is a feature of the plot, which was applied to train the model using the deep learning method. In order to handle the imbalanced data of TWB, the GWAS results of TWB are illustrated with a Manhattan plot after data generation using SMOTE, and then, the similarity was matched with the Manhattan plot of TWB using the SAIGE statistical method for data imbalance processing and the UKB Manhattan plot.

Image processing and image recognition have been extensively studied and applied in biomedicine, such as skeleton using a de-noising filter and image smoothing for retinal images. In addition, the same techniques have been used to develop medical image processing libraries, identify diseases, and determine whether organs are normal (Smistad et al., 2015; Widodo et al., 2020). The results of this study show that the use of a median filtering can remove noise and preserve the features of Manhattan plots. In addition, the image features of the Manhattan plot can be enhanced by adding the dilation process, which increases the classification basis of the recognition system of the trained model. Moreover, in this study, the correctness of the model was generally the highest when the parameter steps_per_epoch was set to 2 or 3. The setting of validation_steps = 4 can obtain a good training model for image recognition and significantly reduce the computation time of the training model.

One of the objectives of this study was to use a TWB data set with a relatively of a small sample size, which after being generated from SMOTE data, can have the same effect as the UKB data set with a relatively larger sample size to identify SNPs associated with genetic diseases. This study used TW-SMOTE with a mixed ratio of a median filter (M) + dilation (D) and model parameters set to steps_per_epoch = 2 and validation_steps = 4 as the training model, and model prediction using the UK-SAIGE Manhattan plot for dilation (D). TW-SMOTE_MD-MIX_ and UK-SAIGE were used for image recognition, and it was found that the HPT and IBS prediction models showed more than 90% correctness (TW-SMOTE_HPT-MD-MIX_, similarity = 0.9719 and TW-SMOTE_IBS-MD-MIX_, similarity = 0.6197, Supplementary Table S5). Furthermore, TW-SMOTE_AST-MD-MIX_ could not be correctly recognized, which may be due to a racial difference in AST, and resulted in different features in the TWB and UKB Manhattan plots (Supplementary Table S5).

Another objective of this study was to generate TWB data through SMOTE, which can show the same effect as other statistical methods (i.e., SAIGE) when handling the data imbalance problem. In this manner, SNPs associated with genetic diseases may be uncovered. Finally, this study determined the optimal data generation ratios (i.e., 0.005 for HPT, 0.03 for IBS, and 0.001 for AST) for three diseases (i.e., HPT, IBS, and AST) through TWB data imbalance. According to the results of this study, the combination with the best performance was the SMOTE proportional mixture (i.e., TW-SMOTE_HPT-MD-MIX_, similarity = 0.9719 and TW-SMOTE_IBS-MD-MIX_, similarity = 0.6197), which used median filtering to remove noise and preserve the image features in the Manhattan plot, and dilation to process the enhanced image features. In addition, the correctness of the model was generally the highest when the parameter steps_per_epoch was set to 2 or 3, good models could be obtained when validation_steps = 4 was applied, and the computing time of the training model could be simultaneously and significantly reduced.

Our study uses the data processing method and deep learning image recognition to deal with the problem of data imbalance. In addition to the statistical method (i.e., SAIGE), our study can be used as another option for researchers to deal with data imbalances. SMOTE may generate too many similar data because of insufficient human data, so it may violate the assumption that the data must meet the independence required in the GWAS analysis. Therefore, we chose diseases with more cases in TWB, namely, AST, HPT, and IBS. In addition, our study selected three diseases with different case–control ratios as the exploration of SMOTE when generating data.

One of the purposes of this study was to use a deep learning image recognition system to identify and compare Manhattan plots from TW-SMOTE and TW-SAIGE to prove the same effect of SMOTE to SAIGE. The other purpose of this study was to evaluate whether the processing of technical data can be equivalent to the use of data plots with a relatively large UKB sample size. Consequently, we used a deep learning image recognition system to identify and compare TWB with relatively few subjects and use SMOTE to generate data (i.e., TW-SMOTE), which can achieve the same results as the UKB (i.e., UK-SAIGE), with more subjects to explain genetic diseases. However, some genetic disorders are more likely to occur among people who trace their ancestry to a particular geographic area (Wright Willis et al., 2010; Flanagin et al., 2021). The principal component analysis (PCA) method is considered to be a very useful method to adjust population stratification in large-scale genetic data analysis (Price et al., 2006; Liu et al., 2013). In our subsequent extended research, our genetic data can be analyzed by PCA to adjust for genetic differences between ethnic groups, and then the Manhattan plot can be drawn. This step can adjust the population stratification for possible ethnic differences in genetic analysis, and the results can help improve the accuracy of deep learning image recognition system.

Our research used a data processing method in TWB data, i.e., SMOTE (named TW-SMOTE), to deal with the problem of imbalance like the traditional statistical method, i.e., SAIGE (named TW-SAIGE). We constructed Manhattan plots from the TW-SMOTE and TW-SAIGE data. Then, we used a deep learning image recognition system to identify and compare the Manhattan plots from TW-SMOTE and TW-SAIGE. Finally, we used the image recognition results to determine our data processing method and achieve the same effect as the statistical method SAIGE to address data imbalance. This study used the Manhattan plot as the basis for image recognition and applied TWB to identify the optimal ratio of data generation for three diseases (HPT, IBS, and AST). The processing of technical data can be equivalent to the use of data plots with a relatively large UKB sample size and achieve the same effect as the statistical method SAIGE to address data imbalance.

## 5 Summary

Our study used a deep learning image recognition system to identify TW-SMOTE and found TW-SMOTE can achieve the same results as that of SAIGE and UK Biobank (UKB). The processing of the technical data can be equivalent to the use of UKB data plots with a relatively large sample size and achieve the same effect as the statistical method SAIGE to address data imbalance.

## Data Availability

The original contributions presented in the study are included in the article/Supplementary Material, and further inquiries can be directed to the corresponding author.
